# Hexyl-Substituted Oligoselenophenes with Central Tetrafluorophenylene Units: Synthesis, Characterisation and Application in Organic Field Effect Transistors^a^

**DOI:** 10.1002/marc.200800449

**Published:** 2008-10-15

**Authors:** David J Crouch, Peter J Skabara, Martin Heeney, Iain McCulloch, David Sparrowe, Simon J Coles, Michael B Hursthouse

**Affiliations:** 1 WestCHEM, Department of Pure and Applied Chemistry, University of StrathclydeGlasgow G1 1XL, UK; 2 School of Chemistry, University of ManchesterManchester M13 9PL, UK; 3 Department of Materials, Queen MaryUniversity of London, London E1 4NS, UK; 4 Department of Chemistry, Imperial College LondonLondon SW7 2AZ, UK; 5 MERCK Chemicals Ltd.Southampton SO16 7QD, UK; 6 Department of Chemistry, University of SouthamptonSouthampton SO17 1BJ, UK

**Keywords:** conjugated polymers, crystal structure, OFETs, selenium, synthesis

## Abstract

A series of selenophene oligomers incorporating conjugated fluorinated phenylene units have been synthesised as potential semiconductor materials for organic field-effect transistors (OFETs). X-ray crystallography shows that the molecules are held in close proximity by several short intermolecular contacts, making them ideal candidates for OFET applications.

## Introduction

**Figure 1:**
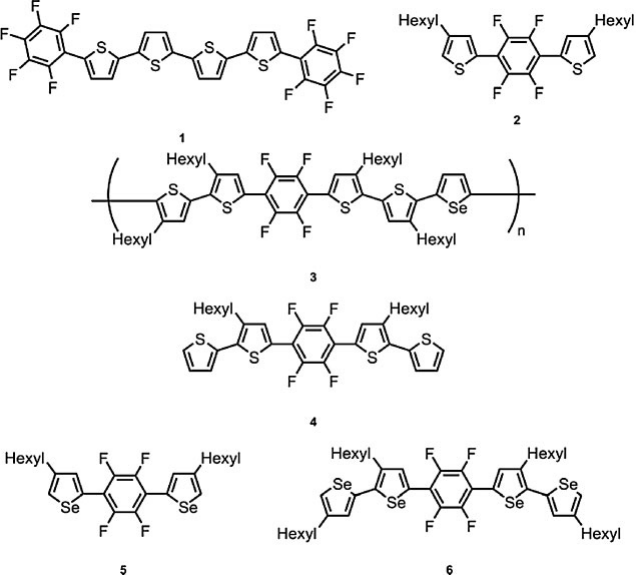
Chemical structures of materials 1–6.

Functionalised oligothiophenes are studied intensively owing to their potential use in a variety of devices such as organic field-effect transistors (OFETs),^1^–^4^ photodiodes,^5^–^7^ light-emitting diodes,^8^–^10^ and their integrated devices.^11^ Work on two important characteristics of organic FETs, namely the on/off ratio and the carrier mobility, has shown that these properties can be optimised by molecular engineering or by controlling the conditions under which well-ordered molecular assemblies can be fabricated.^12^ Fluorinated conjugated oligomers have been of recent topical interest. For example Facchetti et al.^13^ have demonstrated that structures such as **1** are excellent OFET materials. Incorporating fluorinated units into such molecular architectures serves to lower both the LUMO and HOMO energies of the materials and also facilitates the planarisation of **1** (Figure 1). Lowering of the HOMO energy level is a prerequisite in obtaining oxidative stability in thiophene containing polymers.^14^

We recently reported a series of compounds (**2**–**4**) bearing a central tetrafluorophenylene unit.^15^, ^16^ The non-covalent interactions of the fluorine atoms with components on adjacent thiophene rings result in highly planar structures in the solid state, together with excellent solubility of the oligomers and corresponding polymers in common organic solvents. The hybrid oligoselenophene analogue **3** shows p-type conductivity and mobilities that are larger than that of the corresponding pure oligothiophene species.^15^, ^16^ This may be due to the larger and more polarisable selenium atom increasing intermolecular interactions between oligomer backbones. A similar increase in electron rather than hole mobility has been reported by Kunugi et al.^17^ for a biselenophene system, while [1[benzoselenopheno[3,2-*b*[[1[benzoselenophene small molecules exhibit excellent stability in FET devices.^18^ These results imply that poly and oligoselenophenes are excellent candidates for high performance semiconductors.^19^–^21^

Herein, we report the characteristics of OFETs fabricated from fluorinated phenylene–selenophene oligomers.

### Synthesis

**Scheme 1:**
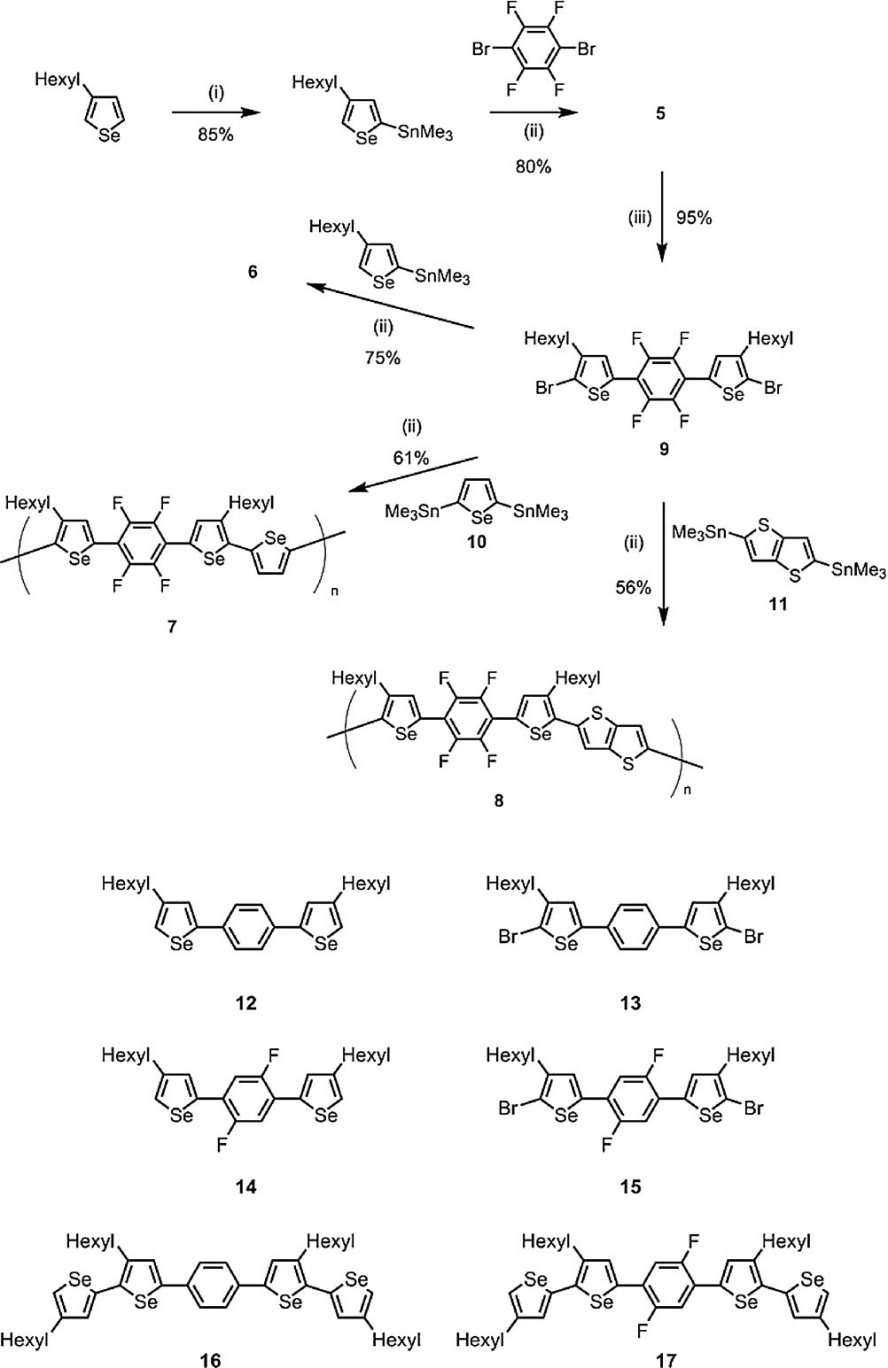
Reagents and conditions: (i) *n*-BuLi then Me_3_SnCl, THF, −80 °C; (ii) tris (dibenzylideneacetone)dipalladium (0), fluorobenzene, tri-*O*-tolylphosphine; 165 °C and (iii) NBS, acetic acid, CHCl_3_, rt.

Compounds **5** and **6** and oligomers **7** and **8** were prepared according to Scheme 1. Unlike the thienyl series, the reaction of (4-hexylselenophen-2-yl)lithium with hexafluorobenzene^15^, ^16^ failed to yield the triaryl derivative **5**. The synthesis requires the conversion of 3-hexylselenophene^22^ to 2-trimethylstannyl-4-hexylselenophene (85% yield), followed by Stille coupling with 1,4-dibromo-2,3,5,6-tetrafluorobenzene (80%, Scheme 1). Bromination of **5** was achieved in ca. 95% yield using *N*-bromosuccinimide in acetic acid/chloroform, while the pentaaryl analogue **6** was obtained by palladium catalysed Stille coupling with 2-trimethylstannyl-4-hexylselenophene (75%). In order to assess the properties of macromolecular analogues, we prepared **7** and **8** via palladium catalysed coupling of the dibromo derivative **9** and the stannylated compounds **10**^23^ and **11**,^24^ respectively. The resulting materials were purified using soxhlet procedures with a range of solvents (acetone, methanol to extract by-products and starting materials, dichloromethane then chloroform to isolate oligomeric fractions). Only oligomers **7** and **8** gave reasonable quality films upon solution deposition (spin coating) and the materials were assessed as p-type semiconductors in organic field effect transistors (*vide infra*).

Using similar synthetic methodology to Scheme 1, compounds **12**–**17** were prepared. Full experimental details for these materials are given in the Supporting Information section, along with analytical data. The materials were synthesised to compare the effect of the fluorine groups in field effect transistors. However, polymer analogues of **13** and **15** produced very poor films and the device characteristics of OFETs prepared from these materials were not sufficiently promising to study these materials further.

### X-Ray Crystallographic Studies

**Figure 2:**
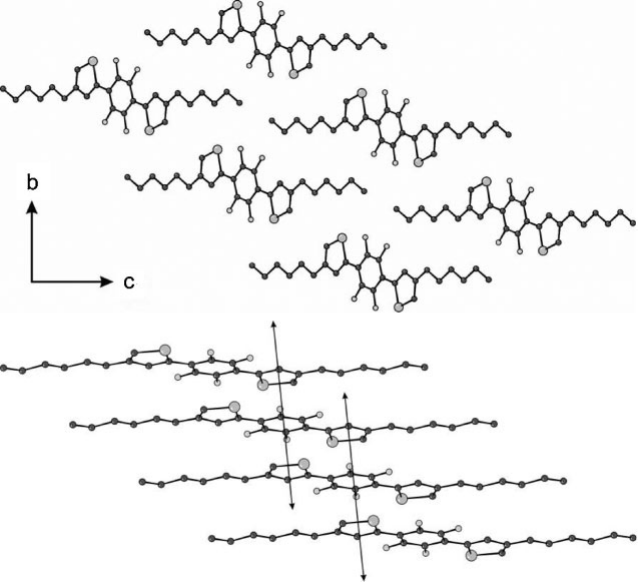
(Top) stacks of 5 propagating along the axis a (into the plane of the graphic). (Bottom) view within the stack, showing intermolecular *π*–*π* contacts.

X-ray crystallographic studies reveal the molecular conformation adopted in the solid state, as well as the identification of possible intermolecular interactions in thin films. Compound **5** was recrystallised from acetone and, as observed in the case of the thiophene analogues,^15^, ^16^ Se–F intramolecular close-contacts (2.749(9) Å) and H–F bonds (2.202(6) Å) are seen to planarise the conjugated segment of the molecule (see Supporting Information for experimental). The molecules form stacks (Figure 2 (top)) with intermolecular ring–ring contacts of 3.62 Å. The exact nature of the *π*–*π* associations can be seen in Figure 2 (bottom) which shows the interaction of the tetrafluorophenylene unit with two selenophene heterocycles. This slip-stack, alternate arrangement has been observed previously^15^, ^16^ and is also well-known for benzene–perfluorobenzene cocrystals,^25^–^27^ in which the tetrafluorobenzene ring has inversed electron density distribution and participates in electrostatic interactions with electron-rich aromatic rings.

### Molecular Weight and Absorption Studies of 7 and 8

The isolated fractions of **7** and **8** are readily soluble in chlorinated solvents. From the soxhlet extraction procedure, dichloromethane and chloroform fractions of **7** corresponded to 3–4 repeat units (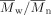 = 1.64) and 7–8 repeat units (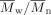 = 1.28), respectively. In the case of **8**, lower molecular weight material was obtained (CH_2_Cl_2_: DP = 2.5, 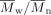 = 1.22; CHCl_3_: DP = 3.5, 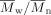 = 1.32). Low molecular weights for such structures are not uncommon.^16^ It is likely that solubility is a major factor, with higher molecular weight fractions being intractable by the purification route adopted. The difference in the degree of polymerisation for **7** and **8** has a direct bearing on their absorption spectra and therefore the mean conjugation length of the macromolecules. Solution state spectra for oligomers **7** and **8** (CHCl_3_ fractions) give optical band gaps of 1.89 eV (**7** *λ*_max_ = 470 nm) and 2.18 eV (**8** *λ*_max_ = 450 nm), respectively. In the solid state, **7** exhibits fine structure in the absorption spectrum, with the longest wavelength absorption maximum at 588 nm. For **8**, the spectrum consists of a single, broad band with a maximum at 497 nm.

### OFET Characteristics

**Figure 3:**
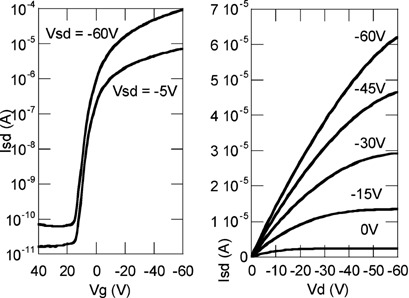
Transfer (left) and output (right) characteristics for oligomer 8.

Transistors employing the chloroform fractions of oligomers **7** and **8** were fabricated by spin coating chlorobenzene solutions onto bottom gate, bottom contact substrates and measured as previously described.^15^, ^16^ Devices were annealed under nitrogen for 10 min at 100 °C prior to measurement. Both oligomers exhibited classical p-type behaviour under negative gate bias. The transfer and output characteristics for oligomer **8** are shown in Figure 3. The oligomer has a saturated field effect mobility of 3 × 10^−3^ cm^2^ · V^−1^ · s^−1^ and a current modulation of greater than 10^6^. The transistor has a relatively high turn-on voltage of +15–20 V, as has been often observed in other electron-rich thiophene^14^, ^24^ and selenophene polymers.^28^ However, the factors influencing the turn-on voltage are not fully understood, with identical polymers demonstrating large variations in turn-on voltage from positive^24^ to negative^29^ for seemingly similar devices structures. It appears that the turn-on voltage is not an intrinsic property of the semiconductor itself but can be influenced by a number of factors including device architecture, dielectric interface and thermal processing history.

Oligomer **7** has a saturated field effect mobility of 2 × 10^−5^ cm^2^ · V^−1^ · s^−1^ and a current modulation of 10^2^. It is likely that the orientation of the alkyl side chains is different for the two oligomer backbones, if they both adopt the most energetically favourable all-trans configurations of the selenium and sulphur atoms. This would result in different packing modes for the oligomer chains, which may partially explain the different performance. Oligomer **8** is a promising candidate for further optimisation. In particular, the quality of the films spun from both oligomers is poor, most likely due to the low molecular weights. An increase in the length of the solubilising alkyl chains should result in higher molecular weight polymers and higher device performance.^30^ Thiophene containing analogues of **7** have given higher values for hole mobility.^16^ Since mobilities are usually greater for the heavier chalcogen in thiophene/selenophene analogues, we assume that the inferior film morphology is responsible for the reduced mobility in **7**.

## Conclusion

In summary, we have synthesised a series of oligoselenophenes bearing a central tetrafluorophenylene core and shown the effect of rigidification through H…F and Se…F contacts by X-ray crystallographic and absorption studies. The oligomer **7** has been prepared via chemical coupling and shows clear evidence of high order and planarity in the solid state. The excellent solubility in common solvents enables deposition of active films by solution processing techniques. Preliminary results on hole mobility measurements for spin-coated oligomers **7** and **8** give values of 2 × 10^−5^ and 3 × 10^−3^ cm^2^ · V^−1^ · s^−1^, respectively. These results were obtained without any transistor optimisation (which would be expected to improve the charge carrier mobility significantly), and are encouraging for chromophores incorporating the diselenyltetrafluorophenylene core. Further studies are underway to optimise the film forming properties and hole mobilities of these materials.

## Notes

Detailed facts of importance to specialist readers are published as ”Supporting Information”. Such documents are peer-reviewed, but not copy-edited or typeset. They are made available as submitted by the authors.
