# Supplementary material for: Hexyl-Substituted Oligoselenophenes with Central Tetrafluorophenylene Units: Synthesis, Characterisation and Application in Organic Field Effect Transistors
Source: Macromol Rapid Commun. 2008 Oct 15;29(22):1839–43. doi: 10.1002/marc.200800449 (PMC3810712; doi:10.1002/marc.200800449)
Supplement: Supplementary file 1 [file marc0029-1839-sd1.pdf]

Copyright WILEY-VCH Verlag GmbH & Co. KGaA, 69469 Weinheim, Germany, 2008.

Supporting Information for *Macromol. Rapid Commun*., 2008, 29, 1839.

Hexyl-Substituted Oligoselenophenes with Central Tetrafluorophenylene Units: Synthesis, Characterisation and Application in Organic Field EffectTransistors

David J. Crouch, Peter J. Skabara,* Martin Heeney, Iain McCulloch, David Sparrowe, Simon J. Coles, and Michael B. Hursthouse

Introduction

In this supplement, we provide experimental procedures for the synthesis of the monomers and oligomers.

General

Unless, otherwise stated all reactions were performed under an inert atmosphere of dry nitrogen using standard Schlenk techniques. All glassware was flamed dried under vacuum prior to use. All solvents and reagents were purchased from Sigma-Aldrich chemical company and used as received. Microwave assisted syntheses were carried out using a Biotage Initiator Sixty EXP Microwave System. UV/vis spectra were recorded using a Unicam UV-300 Spectrophotometer in the range 210-1100 nm, using a 1 cm path length quartz cell. Mass spectra were recorded on a Kratos concept 1S instrument. Infrared spectra were recorded on a Specac single reflectance ATR instrument (4000-400cm^{-1}, resolution 4cm^{-1}). Elemental

analysis was performed by the University of Manchester micro-analytical laboratory. Melting points were recorded on a Gallenkamp melting point apparatus and are uncorrected. 2,5-Bis(trimethylstannyl)selenophene[1] **10** and 2,2'-bis(trimethylstannyl)thieno[3,2-b]thiophene[2] **11** were prepared according to literature procedure.

Crystal data for compound 5

$C_{26}H_{30}F_4Se_2$, Triclinic, *P-1*, *a* = 5.3882(3), *b* = 10.8855(7), *c* = 11.6244(7)Å, α = 115.884(3), β = 103.172(4), γ = 93.878(4)°, volume = 586.30(6)$Å^3$, 120K, Z = 1, D_c = 1.633Mg/m^3, μ = 3.197mm^{-1}, θ_{max} = 27.50°, 12155 measured & 2676 unique (R_{int}=0.0520) reflections, *R1* (obs) = 0.0331 and *wR2* (all data) = 0.0832, ρ_{max}/ρ_{min} = 0.461/-0.594e$Å^{-3}$. Supplementary data in the form of a CIF has been deposited with the Cambridge Crystallographic Data Centre (CCDC 603446).

2-Trimethylstannyl-4-hexylselenophene

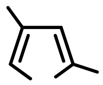

^{t}Butyllithium (28.7 cm^3, 1.7 M, 48.79 mmol) was added dropwise to a stirred solution of 3-hexylselenophene (10 g, 46.47 mmol) in anhydrous tetrahydrofuran (100 cm^3) at -80 °C over a period of 30 min. The resulting yellow solution was heated at -80 °C for 45 min. A solution of trimethyltin chloride (9.85 g, 48.47 mmol) in anhydrous tetrahydrofuran (20 cm^3) was added dropwise at -80 °C over a period of 10 min. The resulting mixture was warmed to room temperature and stirred at this temperature for 3 h. The reaction carefully was poured into water (100 cm^3) and stirred. The solution was extracted with diethyl ether (3 × 100 cm^3). The organic layer was separated and washed with saturated sodium bicarbonate solution (100 cm^3), water (100 cm^3) and brine (100 cm^3) before being dried over magnesium sulphate.

Concentration under reduced pressure yielded a pale yellow oil. Kugelrohr distillation under reduced pressure yielded 12.51 g of colourless oil (71%, Bpt 89-93°C @ 2 × 10^{-2} mbar) FT-IR (KBr): 2924-2869 (C-H str), 1501 (Ar C=C str) cm^{-1}; ^{1}H NMR [d_1-$CDCl_3$]: δ = 0.41 (s, 9H, Sn-C**H**$_3$ $^{\mathbf{2}}J(^{1}H–^{119}Sn)$ 59.96 Hz]), 0.98 (t, 3H, C**H**$_3$-R), 1.39 (m, 6H, 3(C**H**)$_2$-R), 1.70 (m, 2H, C**H**$_2$CH$_2$-Ar) 2.68 (t, 2H, C**H**$_2$-Ar) 7.41 (s, 1H, Ar-**H**), 7.89 (s, 1H, Ar-**H**) ppm; ^{77}Se-{^{1}H} NMR (d_1- $CDCl_3$): δ = 671.919 [$^{\mathbf{2}}J(^{77}Se–^{119}Sn)$ 184.46 Hz] ppm; ^{119}Sn-{^{1}H} NMR (d_1-$CDCl_3$): δ = -20.947 ppm; MS (CI): *m/z* = 380(45%, M+H); (Found: C 41.48 H 6.41 Se 21.09 Sn 31.61 %. $C_{13}H_{24}SnSe$ requires C 41.31 H 6.40 Se 20.89 Sn 31.40 %).

1,4-Bis[2-(4-hexylselenyl)]-2,3,5,6-tetrafluorobenzene (5)

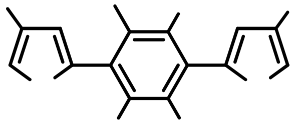

1,4-Dibromo-2,3,5,6-tetrafluorobenzene (0.82 g, 2.64 mmol) and 2-(trimethylstannyl)-4-hexylselenophene (2.00 g, 5.29 mmol) were dissolved anhydrous fluorobenzene (5 cm^3). Tris (dibenzylideneacetone)dipalladium(0) (50.8 mg, 0.0491 mmol) and tri-o-tolylphosphine (61.71 mg, 0.196 mmol) were added and the mixture was irradiated at 165 °C for 10 min. After cooling to room temperature the yellow solution was washed with 2M hydrochloric acid (5 cm^3), water (5 cm^3) and brine (5 cm^3). After drying over magnesium sulphate, the organic extract was concentrated under reduced pressure yielding a yellow powder. Recrystallisation from acetone afforded 1.42 g (93%, Mpt 64-66 °C) of 1,4-bis[2-(4-hexylselenyl)]-2,3,5,6-tetrafluorobenzene *Rf* = 0.63 (hexane): UV/Vis = 291,343,358nm (hexane); FT-IR (KBr): 2961-2894 (C-H str), 1499 (Ar C=C str), 1231(C-F str) cm^{-1}; ^{1}H NMR [d_1-$CDCl_3$]: δ = 0.98 (m, 6H, 2C**H**$_3$-R), 1.38 (m, 12H, 6(C**H**)$_2$-R), 1.69 (m, 4H, 2C**H**$_2$CH$_2$-Ar) 2.62 (t, 4H, 2C**H**$_2$-Ar) 7.40(t, 2H, Ar-**H**), 7.59 (s, 2H, Ar-**H**) 7.65 (s, 2H, Ar-**H**) ppm; ^{19}F-{^{1}H} NMR (d_1-$CDCl_3$): δ = -141.914 ppm [s, 4Ar-F]; ^{77}Se-{^{1}H} NMR (d_1- $CDCl_3$): δ = 620.464ppm; MS

(APCI): *m/z* = 578 (100%, M+H); (Found: C 54.29 H 5.41 F 13.11 Se 27.42 %. $C_{26}H_{30}F_4Se_2$ requires C 54.17 H 5.24 F 13.18 Se 27.39 %).

1,4-Bis{5-[3,4'-dihexyl(2,2'-biselenyl)]}-2,3,5,6-tetrafluorobenzene (6)

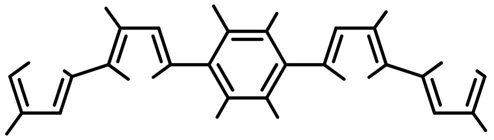

1,4-Bis[2-(5-bromo-4-hexylselenyl)]-2,3,5,6-tetrafluorobenzene (0.15 g, 0.20 mmol) 2-(trimethylstannyl)-4-hexylselenophene (0.16 g, 0.41 mmol) were dissolved in anhydrous fluorobenzene (5 cm^3). Tris(dibenzylideneacetone)dipalladium(0) (3.92 mg, 0.0038 mmol) and tri-o-tolylphosphine (4.77 mg, 0.0152 mmol) were added and the mixture was irradiated at 165 °C for 10 min. After cooling to room temperature the yellow solution was washed with 2M hydrochloric acid (5 cm^3), water (5 cm^3) and brine (5 cm^3). After drying over magnesium sulphate, the organic extract was concentrated under reduced pressure, yielding a viscous yellow oil. Recrystallisation from acetone afforded 0.154 g (45%, Mpt 58-60 °C) of 1,4-bis{5-[3,4'-dihexyl(2,2'-bitselenyl)]}-2,3,5,6-tetrafluorobenzene. The crude product was purified by column chromatography (hexane, silica) yielding 0.289 g (75%, Mpt 58-60 °C) of 1,4-bis{5-[3,4'-dihexyl(2,2'-bithienyl)]}-2,3,5,6-tetrafluorobenzene as orange platelets. *Rf* = 0.44 (hexane): UV/Vis = 294, 403 nm (hexane); FT-IR (KBr): 2922-2856 (C-H str), 1503 (Ar C=C str), 1222 (C-F str) cm^{-1}; ^{1}H NMR (d_1-$CDCl_3$): δ = 0.85 (m, 12H, 4C**H**$_3$-R), 1.35 (m, 24H, 4(C**H**$_2$)$_3$-R), 1.70 (m, 8H, 4C**H**$_2$CH$_2$-Ar) 2.60 (t, 4H, 2C**H**$_2$-Ar) 2.80 (t, 4H, 2C**H**$_2$-Ar) 7.21 (s, 2H, 2Ar-**H**), 7.61 (s, 2H, 2Ar-**H**) 7.84 (s, 2H, 2Ar-**H**) ppm; ^{19}F-{^{1}H} NMR (d_1-$CDCl_3$): δ = -141.620ppm [s, 4Ar-F]; ^{77}Se-{^{1}H} NMR (d_1- $CDCl_3$): δ = 677.741, 622.063ppm; MS (APCI): *m/z* = 1003 (100%, M+H); (Found: C 54.94 H 5.68 F 7.51 Se 31.58 %. $C_{46}H_{58}F_4Se_4$ requires C 55.09 H 5.83 F 7.51 Se 31.49 %).

1,4-Bis[2-(5-bromo-4-hexylselenyl)]-2,3,5,6-tetrafluorobenzene (9)

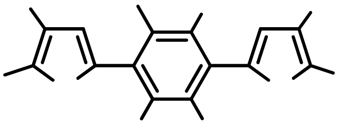

The same procedure was used as for compound **13**. Recrystallisation from chloroform/methanol yielded (97%, Mpt 102-104°C) 1,4-Bis[2-(5-bromo-4-hexylselenyl)]-2,3,5,6-difluorobenzene as a yellow powder. *Rf* = 0.81 (Hexane): UV/Vis = 296,342,357,373 nm (hexane); FT-IR (KBr): 2991-2864 (C-H str), 1488 (Ar C=C str), 1227(C-F str) cm^{-1}; ^{1}H NMR [d$_1$-CDCl$_3$]: δ = 0.87 (m, 6H, 2C**H**$_3$-R), 1.35 (m, 12H, 6(C**H**)$_2$-R), 1.64 (m, 4H, 2C**H**$_2$CH$_2$-Ar) 2.64 (t, 4H, 2C**H**$_2$-Ar) 7.64 (s, 2H, Ar-**H**) ppm; ^{19}F-{^{1}H} NMR (d$_1$- CDCl$_3$): δ = -141.879 ppm [s, 2Ar-F]; ^{77}Se-{^{1}H} NMR (d$_1$- CDCl$_3$): δ = 691.771, 689.684ppm; MS (APCI): *m/z* = 736(87%, M+H); (Found: C 42.41 H 3.81 F 10.49 Br 21.84 Se 21.68 %. C26$H_{28}F_4Se_2Br_2$ requires C 42.53 H 3.84 F 10.35 Br 21.76 Se 21.51 %).

1,4-Bis[2-(4-hexylselenyl)]benzene (12)

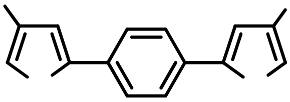

1,4-Diiodobenzene (0.84 g, 2.52 mmol) and 2-(trimethylstannyl)-4-hexylselenophene (2.00 g, 5.29 mmol) were dissolved anhydrous fluorobenzene (5 cm^3). Tris (dibenzylideneacetone)dipalladium (0) (48.5 mg, 0.0468 mmol) and tri-o-tolylphosphine (58.9 mg, 0.187 mmol) were added and the mixture was irradiated at 165 °C for 10 min. After cooling to room temperature the yellow solution was washed with 2M hydrochloric acid (5 cm^3), water (5 cm^3) and brine (5 cm^3). After drying over magnesium sulphate, the organic extract was concentrated under reduced pressure yielding a yellow powder. Recrystallisation from acetone afforded 0.83g (65%, Mpt 50-52 °C) of 1,4-bis[2-(4-hexylselenyl)]benzene as

fine yellow crystals: *Rf* = 0.31 (hexane): UV/Vis = 285, 338 nm (hexane); FT-IR (KBr): 2946-2902 (C-H str) 1503 (Ar C=C str), cm^{-1}; ^{1}H NMR [d_1-$CDCl_3$]: δ = 0.93 (m, 6H, 2C**H**$_3$-R), 1.37 (m, 12H, 6(C**H**)$_2$-R), 1.65 (m, 4H, 2C**H**$_2$CH$_2$-Ar) 2.80 (t, 4H, 2C**H**$_2$-Ar) 7.38 (s, 2H, Ar-**H**), 7.49 (s, 2H, Ar-**H**) 7.53(s, 4H, Ar-**H**) ppm; ^{77}Se-{^{1}H} NMR (d_1- $CDCl_3$): δ = 578.233ppm; MS (APCI): *m/z* = 506 (100%, M+H); (Found: C 62.10 H 6.83 Se 31.45 %. $C_{26}H_{34}Se_2$ requires C 61.90 H 6.79 Se 31.30 %).

1,4-Bis[2-(5-bromo-4-hexylselenyl)]-benzene (13)

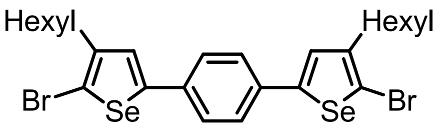

1,4-Bis[2-(4-hexylselenyl)]benzene (0.30 g, 0.59 mmol) was dissolved in a solution of chloroform (20 cm^3) and acetic acid (20 cm^3). N-Bromosuccinimide (0.22 g, 1.22 mmol) was added in one portion at room temperature. The reaction was stirred overnight at room temperature. The yellow solution was poured into 1M hydrochloric acid (50 cm^3) and extracted with chloroform (2 × 100 cm^3). The organic phase was washed with saturated $NaHCO_3$ solution (100 cm^3) and brine (100 cm^3). The extract was dried over magnesium sulphate and concentrated under reduced pressure yielding an orange solid. Recrystallisation from chloroform/methanol yielded 0.294 g (74%, Mpt 130-132 ºC) of 1,4-bis[2-(5-bromo-4-hexylselenyl)]benzene as a cream powder. *Rf* = 0.66 (hexane): UV/Vis = 289,347 nm (hexane); FT-IR (KBr): 2975-2891 (C-H str), 1478 (Ar C=C str), cm^{-1}; ^{1}H NMR [d_1-$CDCl_3$]: δ = 0.98 (m, 6H, 2C**H**$_3$-R), 1.39 (m, 12H, 6(C**H**)$_2$-R), 1.64 (m, 4H, 2C**H**$_2$CH$_2$-Ar) 2.60 (t, 4H, 2C**H**$_2$-Ar) 7.21(s, 2H, Ar-**H**), 7.43 (s, 4H, Ar-**H**) ppm; ^{77}Se-{^{1}H} NMR (d_1- $CDCl_3$): δ = 653.139ppm; MS (APCI): *m/z* = 663(100%, M+H); (Found: C 47.21 H 4.99 Br 24.31 Se 23.71 %. $C_{26}H_{32}Se_2Br_2$ requires C 47.15 H 4.87 Br 24.13 Se 23.85 %).

1,4-Bis[2-(4-hexylselenyl)]-2,5-difluorobenzene (14)

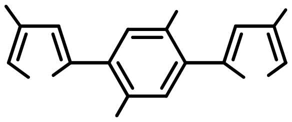

1,4-Dibromo-2,5-difluorobenzene (0.39 g, 1.40 mmol) and 2-(trimethylstannyl)-4-hexylselenophene (1.06 g, 2.81 mmol) were dissolved in anhydrous fluorobenzene (5 cm^3). Tris(dibenzylideneacetone)dipalladium(0) (26.8 mg, 0.0259 mmol) and tri-o-tolylphosphine (32.63 mg, 0.104mmol) were added and the mixture was irradiated at 165 °C for 10 min. After cooling to room temperature the yellow solution was washed with 2M hydrochloric acid (5 cm^3), water (5 cm^3) and brine (5 cm^3). After drying over magnesium sulphate, the organic extract was concentrated under reduced pressure yielding a yellow powder. Recrystallisation from acetone afforded 0.68g (90%, Mpt 85-87 °C) of 1,4-bis[2-(4-hexylselenyl)]-2,5,-difluorobenzene *Rf* = 0.49 (hexane): UV/Vis = 289, 362, 371 nm (hexane); FT-IR (KBr): 2947-2888 (C-H str), 1496 (Ar C=C str), 1228(C-F str) cm^{-1}; ^{1}H NMR [d_1-$CDCl_3$]: δ = 0.98 (m, 6H, 2C**H**$_3$-R), 1.38 (m, 12H, 6(C**H**)$_2$-R), 1.69 (m, 4H, 2C**H**$_2$CH$_2$-Ar) 2.62 (t, 4H, 2C**H**$_2$-Ar) 7.40(t, 2H, Ar-**H**), 7.59 (s, 2H, Ar-**H**) 7.65 (s, 2H, Ar-**H**) ppm; ^{19}F-{^{1}H} NMR (d_1-$CDCl_3$): δ = -120.147 ppm [s, 2Ar-F]; ^{77}Se-{^{1}H} NMR (d_1- $CDCl_3$): δ = 617.542, 615.846ppm; MS (APCI): *m/z* = 543 (100%, M+H); (Found: C 57.91 H 5.91 F 7.14 Se 28.99 %. $C_{26}H_{32}F_2Se_2$ requires C 57.78 H 5.97 F 7.03 Se 29.22 %).

1,4-Bis[2-(5-bromo-4-hexylselenyl)]-2,5-difluorobenzene (15)

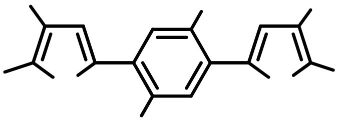

The same procedure was used as for compound **13**. Recrystallisation from chloroform/methanol yielded (93%, Mpt 78-81 ºC) 1,4-bis[2-(5-bromo-4-hexylselenyl)]-2,5-difluorobenzene as a cream powder. *Rf* = 0.77 (hexane): UV/Vis = 287, 370, 380 nm (hexane); FT-IR (KBr): 2984-2893 (C-H str), 1499 (Ar C=C str), 1222(C-F str) cm^{-1}; ^{1}H NMR [d_1-$CDCl_3$]: δ = 0.91 (m, 6H, 2C**H**$_3$-R), 1.38 (m, 12H, 6(C**H**)$_2$-R), 1.63 (m, 4H, 2C**H**$_2$CH$_2$-Ar) 2.62 (t, 4H, 2C**H**$_2$-Ar) 7.25-7.41 (m, 4H, Ar-**H**) ppm; ^{19}F-{^{1}H} NMR (d_1-$CDCl_3$): δ = -120.283 ppm [s, 2Ar-F];^{77}Se-{^{1}H} NMR (d_1- $CDCl_3$): δ = 688.672, 686.561ppm; MS (APCI): *m/z* = 698(75%, M+H); (Found: C 44.83 H 4.31 F 5.47 Br 23.09 Se 22.58 %. C26$H_{32}F_2Se_2Br_2$ requires C 44.72 H 4.33 F 5.44 Br 22.89 Se 22.62 %).

1,4-Bis{5-[3,4'-dihexyl(2,2'-biselenyl)]}-benzene (16)

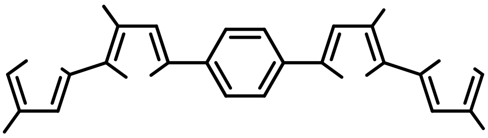

The same procedure was used as for compound **6**. The crude product was recrystallised from acetone yielding (71%, Mpt 49-51 °C) 1,4-bis{5-[3,4'-dihexyl(2,2'-biselenyl)]}-benzene as an orange powder. *Rf* = 0.27 (Hexane): UV/Vis = 321, 383 nm (hexane); FT-IR (KBr): 2977-2894 (C-H str), 1509 (Ar C=C str), cm^{-1}; ^{1}H NMR (d_1-$CDCl_3$): δ = 0.88 (m, 12H, 4C**H**$_3$-R), 1.38 (m, 24H, 4(C**H$_2$**)$_3$-R), 1.70 (m, 8H, 4C**H**$_2$CH$_2$-Ar) 2.64 (t, 4H, 2C**H**$_2$-Ar) 2.81 (t, 4H, 2C**H**$_2$-Ar) 6.97 (s, 2H, 2Ar-**H**), 7.03 (s, 2H, 2Ar-**H**) 7.21 (s, 2H, 2Ar-**H**) 7.61 (s, 4H, Ar-**H**)

ppm; ^{77}Se-{^{1}H} NMR (d_1- $CDCl_3$): δ = 656.661, 579.437ppm; MS (APCI): *m/z* = 935 (100%, M+H); (Found: C 59.37 H 6.84 Se 33.87 %. $C_{46}H_{62}Se_4$ requires C 59.36 H 6.71 Se 33.93 %).

1,4-Bis{5-[3,4'-dihexyl(2,2'-biselenyl)]}-2,5-difluorobenzene (17)

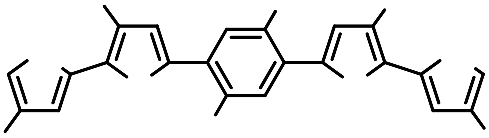

The same procedure was used as for compound **6**. Recrystallisation from acetone afforded (79%, Mpt 67-69 °C) 1,4-bis{5-[3,4'-dihexyl(2,2'-biselenyl)]}-2,5-difluorobenzene as an orange powder. *Rf* = 0.30 (hexane): UV/Vis = 406 nm (hexane); FT-IR (KBr): 2961-2895 (C-H str), 1506 (Ar C=C str), 1228 (C-F str) cm^{-1}; ^{1}H NMR (d_1-$CDCl_3$): δ = 0.90 (m, 12H, 4C**H**$_3$-R), 1.39 (m, 24H, 4(C**H$_2$**)$_3$-R), 1.68 (m, 8H, 4C**H**$_2$CH$_2$-Ar) 2.61 (t, 4H, 2C**H**$_2$-Ar) 2.79 (t, 4H, 2C**H**$_2$-Ar) 7.19 (s, 2H, 2Ar-**H**), 7.39 (t, 2H, Ar-**H**), 7.51 (s, 2H, 2Ar-**H**) 7.63 (s, 2H, Ar-**H**) ppm; ^{19}F-{^{1}H} NMR (d_1-$CDCl_3$): δ = -120.447ppm [s, 4Ar-F]; ^{77}Se-{^{1}H} NMR (d_1-$CDCl_3$): δ = 678.464, 617.542, 615.846ppm; MS (APCI): *m/z* = 970 (100%, M+H); (Found: C 57.26 H 6.31 F 4.08 Se 32.77 %. $C_{46}H_{60}F_2Se_4$ requires C 57.14 H 6.26 F 3.93 Se 32.67%).

Oligomer 7

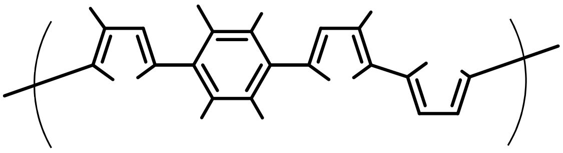

1,4-Bis[2-(5-bromo-4-hexylselenyl)]-2,3,5,6-tetrafluorobenzene (0.05 g, 0.0681 mmol), and 2,5-bis(trimethyltin)selenophene (0.031 g, 0.0681 mmol) were dissolved anhydrous fluorobenzene (5cm^3). Tris (dibenzylideneacetone)dipalladium(0) (1.31 mg, 0.00126 mmol) and tri-o-tolylphosphine (1.59 mg, 0.0152 mmol) were added and the mixture was irradiated at 165 °C for 10 min. The resulting blood red reaction mixture was precipitated in methanol,

and further purified *via* soxhlet extractions using methanol, acetone, isohexane. The resulting solid was dissolved in hot chloroform and filtered. Concentration under reduced pressure afforded a purple powder (0.03 g, 60%); UV/Vis = 300, 469, 586 nm ($CHCl_3$); MS (MALDI-TOF): n = 2,3,4,5 and 6 peaks (1404, 2111, 2807, 3515, 4221) ($CHCl_3$); GPC(PhCl) = M_n = 5.2×10^3; M_w = 6.7×10^3; D = 1.28; (Found: C 48.27 H 4.44 F 10.61 Se 32.41 %. $C_{30}H_{30}F_4Se_3$ requires C 51.22 H 4.30 F 10.80 Se 33.67 %).

Oligomer 8

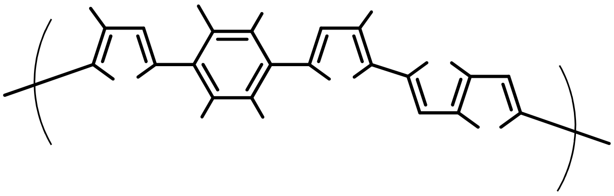

Yield = 72%, UV/Vis = **291, 481, 612** nm ($CHCl_3$); MS (MALDI-TOF): n = 2,3 and 4 peaks (1439, 2130, 2841) ($CHCl_3$); GPC(PhCl) = M_n = 2.6×10^3; M_w = 4.2×10^3; (Found: C 53.11 H 4.38 F 10.21 S 9.21 Se 22.00 %. $C_{32}H_{30}F_4S_2Se_2$ BU requires C 53.93 H 4.24 F 10.66 S 9.00 Se 22.16 %).

[1] D. E. Seitz, S. H. Lee, R. N. Hanson and J. C. Bottaro, *Synth. Commun.* **1983**, *13*, 121.

[2] I. McCulloch, M. Heeney, C. Bailey, K. Genevicius, I. Macdonald, M. Shkunov, D. Sparrowe, S. Tierney, R. Wagner, W. M. Zhang, M. L. Chabinyc, R. J. Kline, M. D. McGehee and M. F. Toney, *Nat. Mater.* **2006**, *5*, 328.
